# Sustained Improvements of Thoracic Hyperkyphosis, Body Dysmorphic Disorder, in a 26-Year-Old Male With Scheuermann’s Disease Using Chiropractic BioPhysics® Spinal Rehabilitation: A Case Report and 28-Month Long-Term Follow-Up

**DOI:** 10.7759/cureus.116012

**Published:** 2026-09-09

**Authors:** Zyrah Giustra, Jason W Haas, Paul A Oakley, Joe Betz, Deed E Harrison

**Affiliations:** 1 Chiropractor, Private Practice, Boise, USA; 2 Research, Chiropractic BioPhysics (CBP) NonProfit, Inc., Windsor, USA; 3 Kinesiology and Health Science, York University, Toronto, CAN; 4 Chiropractic, Private Practice, Newmarket, CAN; 5 Chiropractic, Private Practice, Boise, USA; 6 Research, Chiropractic BioPhysics (CBP) NonProfit, Inc., Eagle, USA

**Keywords:** body-dysmorphic disorder, musculoskeletal rehabilitation, radiography, scheuermann’s disease, thoracic spine

## Abstract

We report sustained improvements in thoracic hyperkyphosis, sagittal balance, and health-related quality of life in a 26-year-old male with Scheuermann’s disease using Chiropractic BioPhysics® (CBP®) rehabilitation with a 28-month long-term follow-up. Scheuermann’s is associated with significant reductions in quality-of-life outcomes and poor psychosocial outcomes. Body dysmorphic disorders and altered self-perception further deteriorate patient-reported outcomes. Reductions in abnormal spine conditions can reduce the likelihood of disability and chronic conditions.

A 26-year-old male with posture deformity and hyperkyphosis had experienced only long-term mild pain and consequences from his abnormal posture. Poor sagittal alignment led to disqualification for enrollment into the United States Coast Guard; he sought treatment in Meridian, Idaho, USA. Physical examination demonstrated altered spine and postural alignment. Treatment was a multi-modal regimen focused on postural muscles, specific spine manipulation directed toward abnormal full-spine alignment, and specific mirror image traction aimed to improve spine integrity by realigning the spine toward a more normal position. Following 36 treatments over three months, all initial tests and outcome measures were repeated. All outcome measures, posture, and radiographic mensuration demonstrated improvement at the conclusion of treatment and were maintained at 28-month re-examination. SF-36 scores improved by the minimal clinically important difference in nearly all categories and were maintained at 28 months.

We report that the CBP^®^treatment approach had a potential positive effect for a single patient with Scheuermann’s kyphosis. This investigation is a single case, and no declarative statements of efficacy are made or implied. Larger controlled studies are necessary to make conclusions regarding Scheuermann's kyphosis with CBP treatment. This study adds to the literature regarding conservative methods of treating postural and spine disorders.

## Introduction

Postural hyperkyphosis and Scheuermann’s disease are conditions that are associated with poor posture, accelerated degeneration and dysfunction, and increased risk of chronic pain and suffering, thus increasing disability risk [1]. It has been known for many decades that low back pain, neck pain, and other musculoskeletal pain are a major factor in poor health-related quality of life (HRQoL) outcomes and are frequently amongst the leading causes of disability globally and contribute significantly to the global burden of disease (GBD) [2,3]. There are fewer investigations of the biopsychosocial impact of abnormal posture due to conditions such as Scheuermann’s disease. Treatment of spine musculoskeletal conditions has been estimated to cost more than $40 billion (USD) annually. These disabilities contribute greatly to years lived with disability (YLDs) and are important topics for biomechanical and biomedical healthcare fields [4]. Recent studies have sought to illuminate treatment options, both conservative and surgically [5].

We present a case documenting the improvements in posture, spine alignment, and psychosocial effects following structural rehabilitation in a 26-year-old male. The patient had desired to become a member of the United States Coast Guard (USCG) and was denied entry due to his postural abnormality [6]. There is a large gap in the literature regarding body dysmorphic disorder, Scheuermann's Disease and service and ability to serve in the medical literature. This denial of service to the USCG caused him great distress and contributed significantly to a reduction in his mental health quality and self-perception of his body, increasing potential for body dysmorphic disorder (BDD). This BDD and worsening posture were worsening his biopsychosocial affect and self-perception. The USCG has a cut point of greater than 40° maximum Cobb angle as disqualifying for service. The patient was very concerned about this disqualification and sought treatment at a spine treatment facility. The diagnosis was made primarily by the visible presence of postural posterior thoracic translation and thoracic hyperkyphosis, and patient discomfort with his posture. 

Abnormal posture and abnormal spine alignment have been shown to contribute to chronic pain and disability. Recent studies have shown effective conservative therapies designed to reduce these abnormalities [7]. Evaluation of the spine alignment requires radiography as the gold standard for biomechanical assessment [8]. The purpose of this case report is to elucidate the treatment of abnormal posture and spine alignment associated with Scheuermann’s kyphosis to improve structure and minimize the psychosocial consequences and BDD that can arise when patients have abnormal spine alignment and visibly poor posture [9]. This case details the history, subjective and objective examination findings and post-treatment improvement with long-term follow-up. This investigation bridges the gap in knowledge and application of diagnostic and therapeutic approaches for treatment. This case is unique because the patient was suffering not primarily from pain, but also from abnormal biopsychosocial impacts. Given this unique case, the patient was selected for publication with the aim of educating radiologists and providers on conservative treatment for those with Scheuermann’s disease.

## Case presentation

Ethical considerations, patient history, examination results and outcome measure findings

The retrospective nature of the study makes Institutional Review Board approval not applicable due to the in accordance with the United States Health and Human Services regulation Common Rule’s exemption 45 CFR 46.104 concerning benign case report investigations of agreeable patients under informed consent. This study’s physician and lead author (Z.G.) obtained informed consent, and patient consent to publish was acquired prior to treatment and publication. The case report does not contain identifiable personal patient data, and the Declaration of Helsinki requirement for further scrutiny is not applicable for this investigation, as no patient identifiers are presented. Data is presented without any personal patient identifiers. This report was conducted with a commitment to ethical integrity, and data was used in a way that poses no risk to the individual patient.

A 26-year-old male presented to a spine rehabilitation facility in Nampa, Idaho, with thoracic hyperkyphosis and a diagnosis of Scheuermann’s disease. This hyperkyphosis is accompanied by the diagnostic requirement of three wedged vertebrae. This diagnosis and postural abnormality disqualified him for USCG enlistment. Thoracic kyphosis with a Cobb angle greater than 40° is considered a disqualifying medical condition for the USCG. Scheuermann’s hyperkyphosis is a rare condition. The patient had had minimal prior treatment for some musculoskeletal pain due to exercise-induced soreness in the mid-back. The prior treatment was a recommendation to take anti-inflammatories when the pain became too intense and to reduce the activities that caused the pain (high-intensity exercise). 

The patient reported minimal mid-back pain (MBP) and low back pain (LBP) and was more concerned about his spinal alignment and posture. He stated his posture appeared to be worsening. He rated his MBP and LBP 1/10 on a Numeric Rating Scale (NRS), where 0 is no pain and 10 is the worst pain possible. He completed an Oswestry Disability Index (ODI) outcome measure to determine his self-rated disability due to LBP. The patient scored 16% (mild disability due to LBP) on a scale of 0-100%, where 0 is no disability, and 100 is complete disability [10]. The patient completed the short-form 36-question (Sf-36) HRQoL survey to determine the impact of his MBP and LBP on his quality of life (QOL) and to assess his self-rated disability [11]. The SF-36 outcome questionnaire provides scaled scores for different HRQoL domains on a scale of 0 to 100, where 0 is the lowest HRQoL, and 100 is the highest. Pre-treatment SF-36 scores below normal limits showed: Bodily Pain (BP) was 37.5%, emotional wellbeing (EWB) was 48.4%, energy/fatigue (EF) was 45.5%, and general health (GH) was 60% (Table 1).

**Table 1:** Radiographic, subjective, and objective outcome measures: pre-treatment findings, results, and follow-up. ARA: Absolute rotational angle; Cobb: Cobb angle; Tz: Sagittal translation (translation in the z-axis); NRS: Numeric rating scale; LBP: Low back pain; MBP: Mid-back pain; ODI: Oswestry disability index; NDI: Neck disability index.

Health Outcome Measure	Ideal Values	Pre-Treatment	Re-Exam	Post-Treatment	Follow-up	Follow-up
Radiographic Mensuration			8 weeks	3 months	10 months	28 months
ARA T1-T12 (°)	44	52.3	59.3	50.9	53.6	41.4
ARA T2-T11 (°)	42	55.5	54.9	43.8	42	52.2
ARA T3-T10 (°)	37	48.5	35.4	34.8	31.4	34.9
ARA T4-L2 (Max ARA) (°)	30	86.7	66.8	51.3	50.7	75
Cobb T1-T12 (°)	20-40	60.4	69.3	57.7	40.7	46.2
Cobb T4-T12 (°)	n/a	72.5	64.1	62.7	56.1	61.6
Cobb T4-L2 (Max Cobb) (°)	n/a	82.4	74	64.6	59	61.6
Tz T12-S1 (mm)	0	-111.3	-77.4	-64.1	-68.6	-87.1
Tz T1-T12 (mm)	0	61.5	62.3	40.3	12.7	62.9
Questionnaire Outcomes						
NRS (LBP)	0	1	0	0	0	0
NRS (MBP)	0	1	0	0	0	1
ODI (%)	0	16	0	0	0	0
SF-36 Energy/Fatigue	68-100	45.5	60	90	90	100
SF-36 Emotional Well-Being	68-100	48.4	60.5	68	68	76
SF-36 Bodily Pain	68-100	37.5	50	100	100	100
SF-36 General Health	68-100	60	60	100	90	75

**Figure 1:**
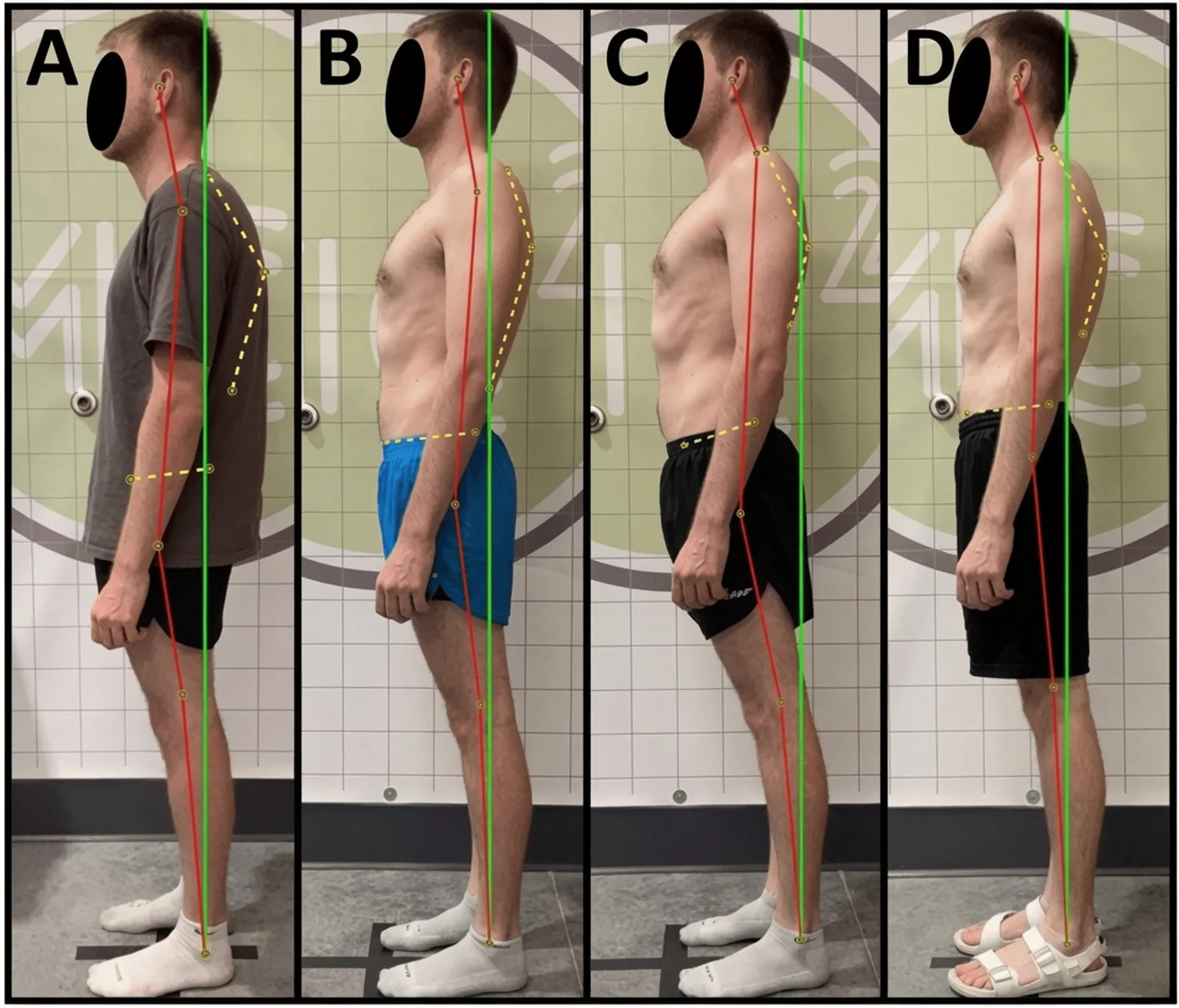
Full spine lateral posture photographs throughout and after treatment. Pre-treatment sagittal posture analysis. A) Pre-treatment, B) Re-exam, c) Post-treatment, and D) Long-term follow-up posture analysis using PostureScreen® Sagittal postural pictures of the patient are presented. The patient was uncomfortable removing his shirt at the initial evaluation due to the thoracic deformity. The green line represents a simple vertical axis plumb line where normal posture would have the major body part centers of mass equal over each other. The exterior auditory meatus, acromioclavicular joint, lateral center of the acetabulum and the lateral malleolus of the ankle would be expected to be near vertical over each other in ideal posture. The red lines represent the actual sagittal posture of the patient. The yellow circles with the black ring inside are anatomical landmarks made to analyze the patient’s posture, and the yellow dotted lines show the approximate thoracic kyphosis and pelvic tilt measurements. The patient first presented with a posture that was "collapsed" and he felt he "couldn't stand up straight", and felt very uncomfortable about exposing his spine. The 2nd, 3rd, and 4th images were taken following treatment; he reported much better ability to stand up straight without effort, felt more postural confidence and didn't feel like he was "collapsing" as he did at the initial exam. He was no longer uncomfortable taking off his shirt and exposing his torso.

Posture analysis was performed using PostureScreen® Mobile Posture Analysis (PostureCo, Inc., Trinity, FL, USA) [12,13]. Pre-treatment sagittal posture analysis showed anterior pelvic and head translation (+TzP, +TzH), posterior thorax translation (-TzT), and increased thorax flexion (+RxT) with anterior sagittal balance (Figure 1).

**Figure 2:**
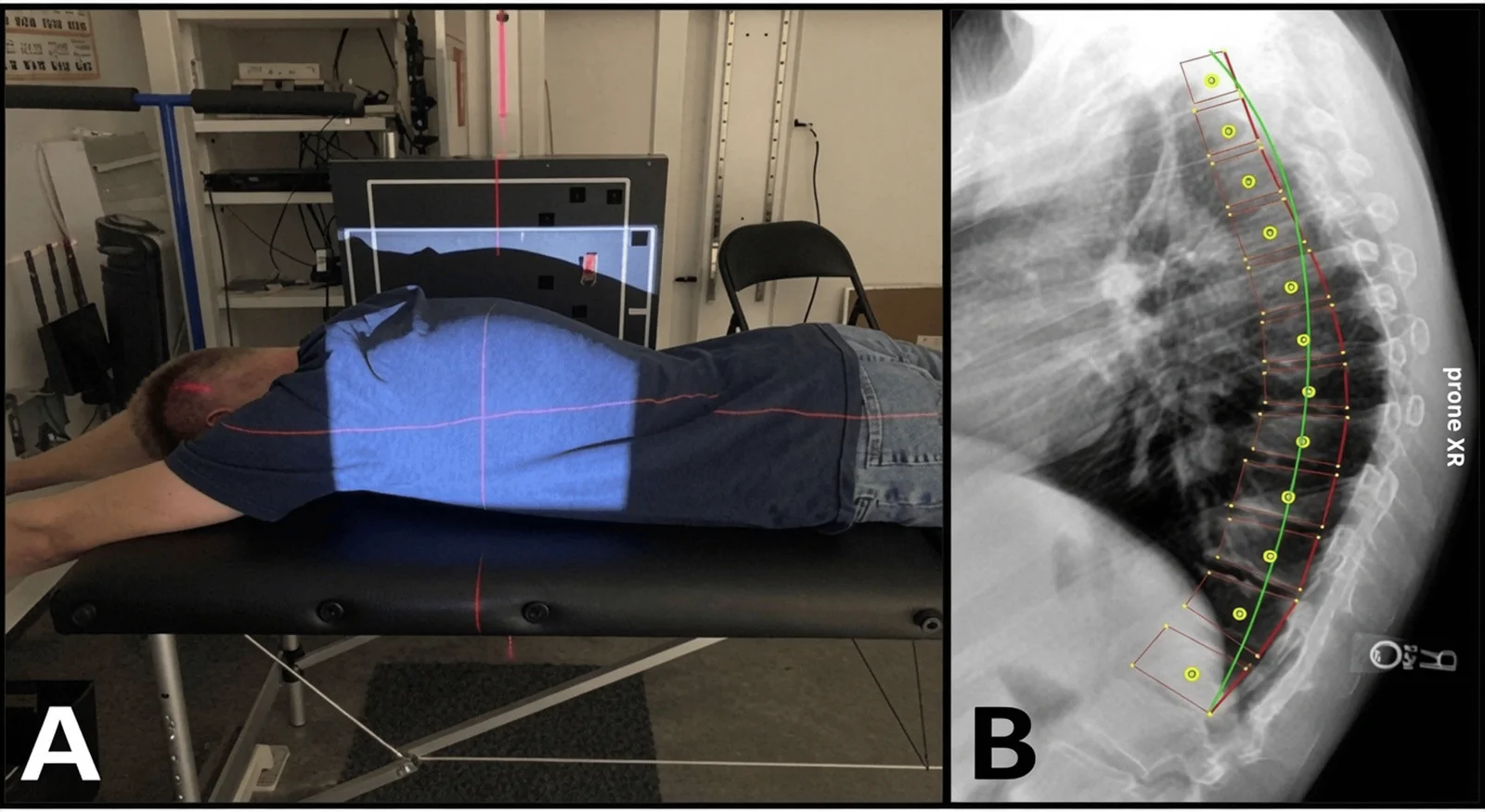
Thoracic spine flexibility prone radiograph positioning and results. A-B) Prone postural correction potential position showing lateral thoracic radiograph position and image results. The patient was placed in the prone position to ascertain the potential correction and overall flexibility of the thoracic spine in the position that reduced the spine deformity. The light represents the frame of the radiograph and the patient was attempting to flatten the thoracic spine as his muscles would allow. The results are then applied to the therapeutic treatment regimen of MI® exercises, traction, and postural SMT.

**Figure 3:**
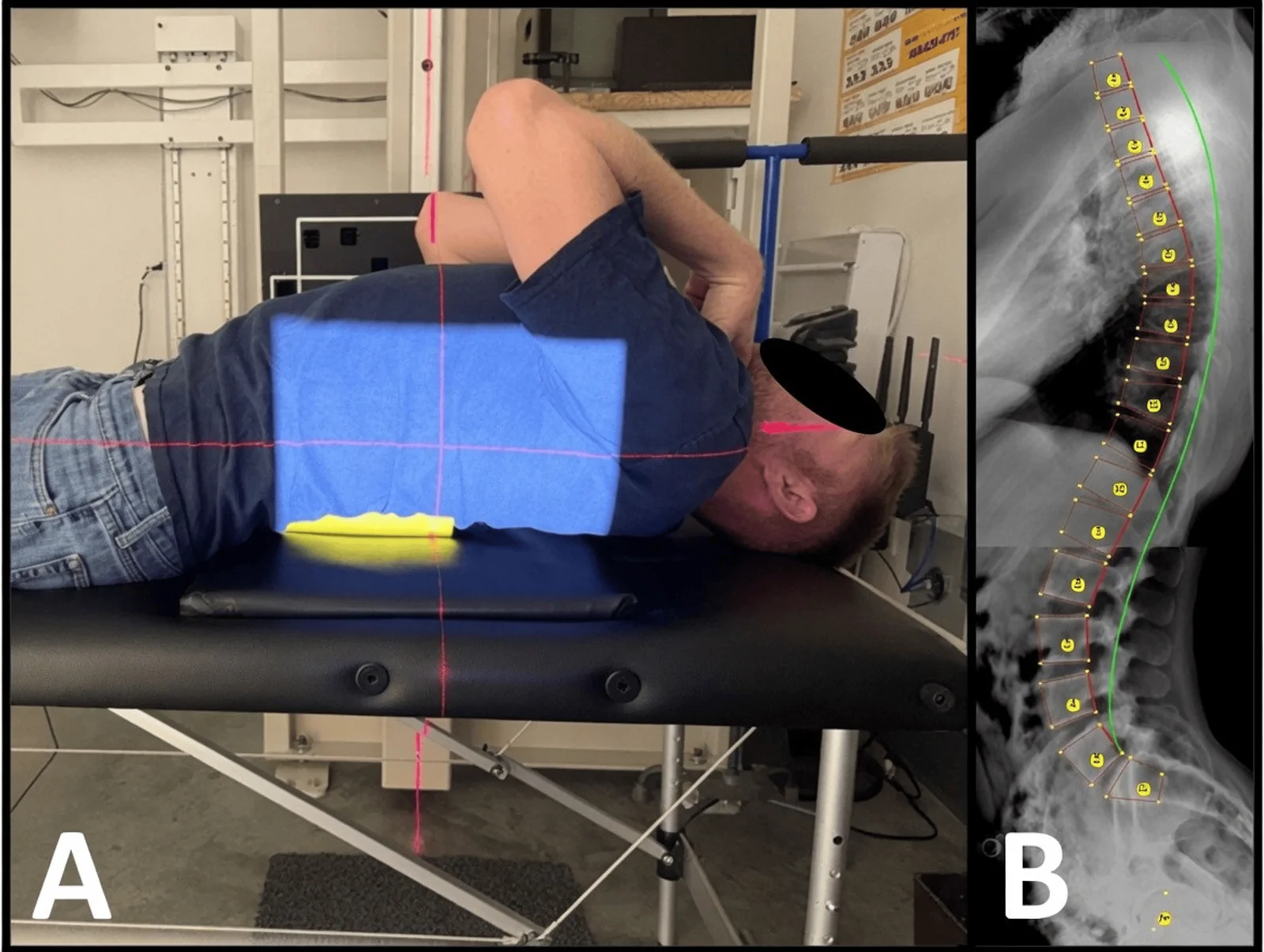
Thoracolumbar spine flexibility supine radiograph with Denneroll(r) and results. (A) Supine radiograph position for thoracic spine flexibility and (B) image results (A) Supine correction potential radiograph with the patient placed in the prone position, with a small, firm foam Denneroll® wedge (TDSO) placed at the apex of the abnormal thoracic kyphosis to induce anterior translation and extension of the thoracic spine. (B) Lateral thoraco-lumbopelvic upright radiograph where the PostureRay® digitization software results are presented as red squares at each margin of the vertebral bodies, as measured by the yellow dots at the anterior-superior, posterior-superior, anterior-inferior, and posterior-inferior points.

**Figure 4:**
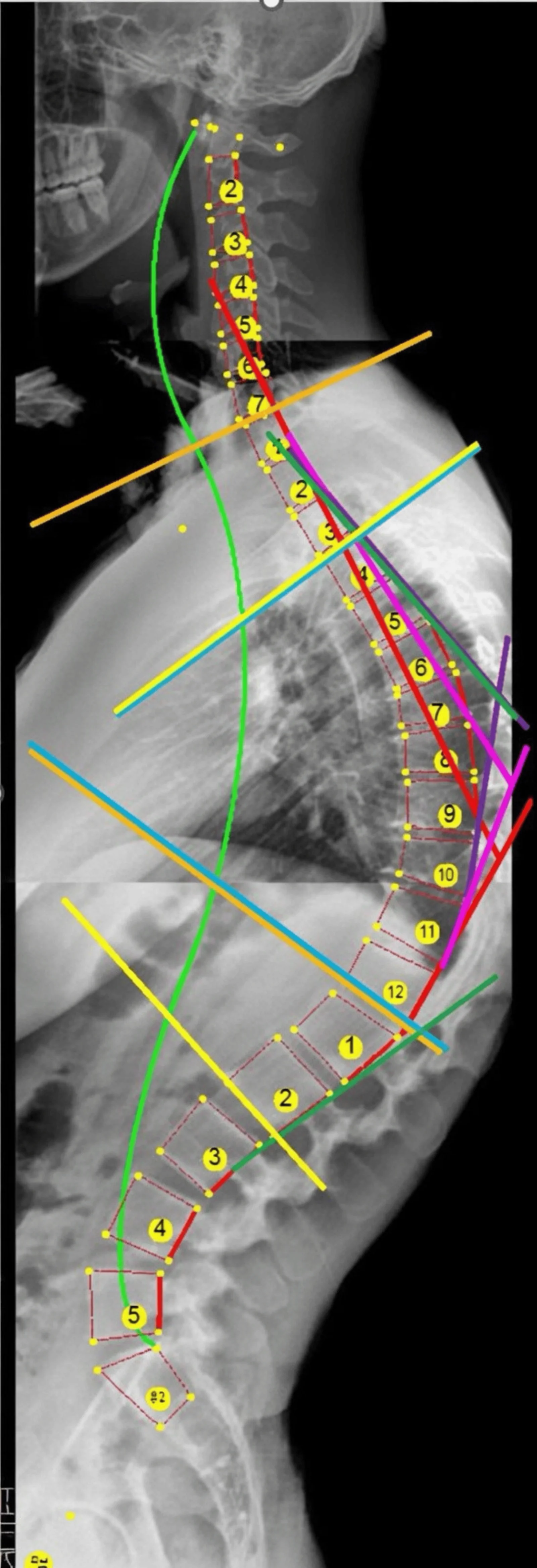
Pre-treatment lateral full spine radiograph with lines of mensuration and explanation. The full-spine lateral radiograph with color-coded mensuration lines. The green continuous line with 3 curves represents a normal, ideal sagittal spinal alignment and posture. The red lines represent the actual C2-S1 posterior tangent lines of the patient. The black numbers in yellow circles are the numbered cervical, thoracic, lumbar, and sacral vertebrae. The yellow dots represent the marked C1 anatomical landmarks, anterior superior and inferior and posterior superior and inferior aspects of C2-S1 and hip anatomical landmarks. The orange lines through the vertebral endplates are the Cobb T1-T12 angle; the yellow lines through the vertebral endplates are the Cobb T4-L2 angle; the blue lines through the vertebral endplates are the Cobb T4-T12 angle; the red elongated posterior tangent lines are the ARA (Absolute Rotation Angle) T1-T12; the chartreuse posterior tangent lines are the ARA T2-T12; the purple posterior tangent lines are the ARA T3-T10; and the green posterior tangent lines are the ARA T4-L2; the red lines are the ARA T1-T12.

**Figure 5:**
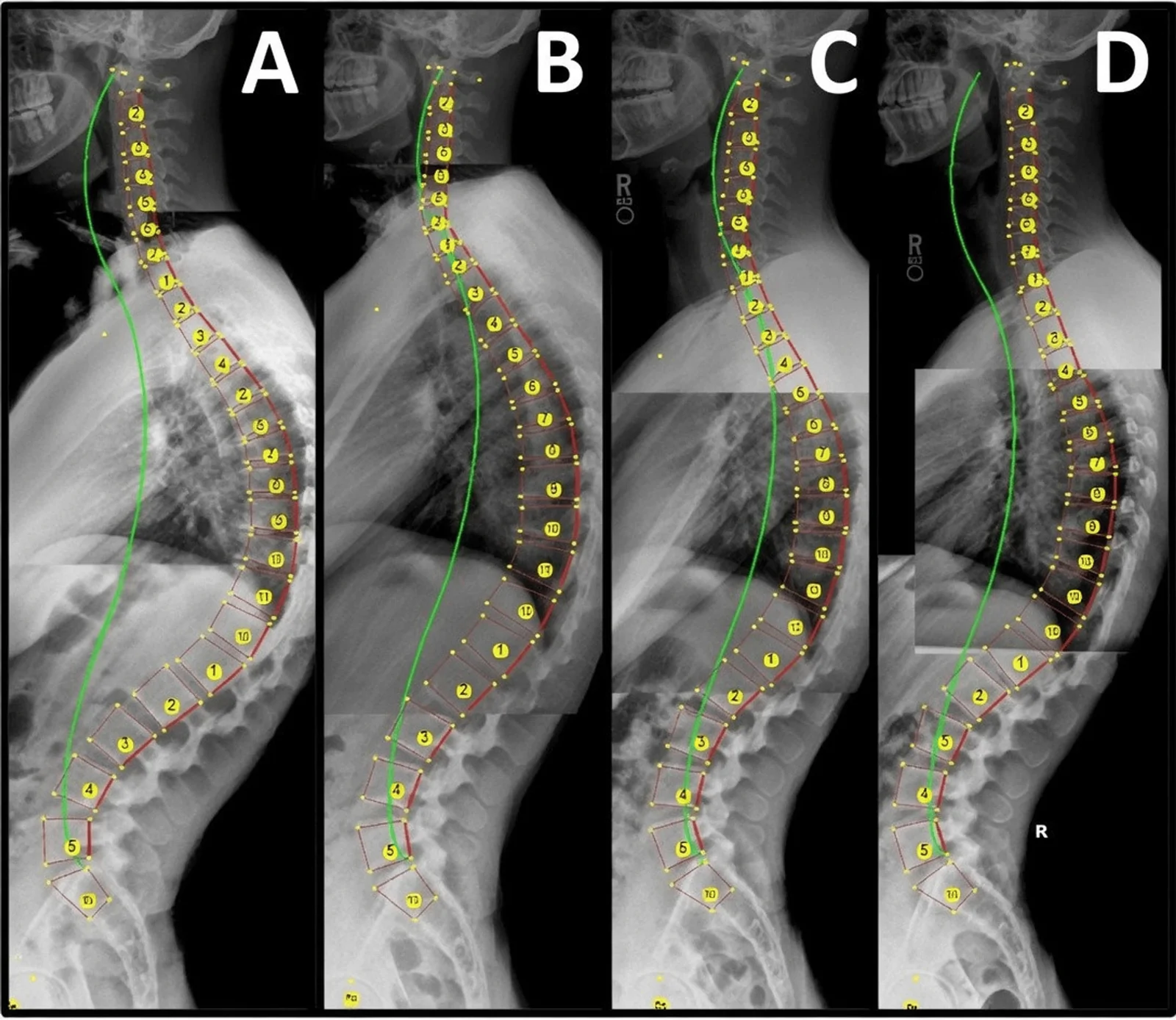
Full spine sagittal spine radiographs before, after and long-term follow-up with lines of mensuration. A) Pre-Treatment, B) Re-Exam, C) Post-Treatment, and D) Long-Term Follow-Up Sagittal Full-Spine (Stitched Sectionals) Radiographs. The green line represents a normal, ideal sagittal spinal alignment and posture. The red lines represent the C2-S1 posterior tangent lines of the patient. The black numbers in yellow circles are the numbered cervical, thoracic, lumbar, and sacral vertebrae. The yellow dots represent the marked C1 anatomical landmarks, anterior superior and inferior and posterior superior and inferior aspects of C2-S1 and hip anatomical landmarks.

Radiographic analysis was performed using PostureRay® digital mensuration software (PostureCo, Inc., Trinity, FL, USA) [14]. Pre-treatment sagittal radiographic analysis confirmed the Scheuermann’s disease diagnosis and showed posterior sagittal imbalance and hyperkyphosis of the thoracic spine. Recorded were the following measurements: absolute rotational angle (ARA) of T2-T11 measuring 55.5° (ideal is 42°), ARA T4-L2 (max ARA) measuring 86.7° (ideal is 30°), Cobb angle from T4 to T12 (Cobb T4-T12) measuring 72.5°, Cobb T4-L2 (max Cobb) measuring 82.4°, sagittal translation of T12 with respect to S1 (Tz T12-S1) measuring -111.3 mm (ideal is 0 mm), and Tz T1-T12 measuring 61.5 mm (ideal is 0 mm) (Figures 2-3A, Table 1) [15-17]. Correction potentialTM radiographs were taken in the prone position (Figure 2) and in the supine position with a Thoracic DennerollTM Spinal Orthotic (TDSO) (DennerollTM Spinal Orthotics, New South Wales, Australia) placed at the mid-to-lower thoracic spine to create thoracic extension (Figure 3). Both correction potential™ radiographs showed reduction in thoracic hyperkyphosis and improvement in sagittal balance, indicating flexibility of the thoracic spine (Figure 2B, Figure 3B). Figure 4A-4D presents the overall measurements of the lateral full spine radiograph, including the RRA, ARA and Cobb analysis of the patient [19] for each examination. The measurements of the Cobb and Harrison Posterior Tangent methods are presented on a single full spine lateral radiograph in Figure 5. These points allow the Harrison Posterior Tangent Method (HPTM) and Cobb analysis of any combination of posterior tangents for each RRA, total ARA, and endplate tangents [18] (Figure 5).

These points allow the Harrison Posterior Tangent Method (HPTM) and Cobb analysis of any combination of posterior tangents for each RRA, total ARA, and endplate tangents [18] (Figure 4). Figure 5A-5D presents the overall measurements of the lateral full spine radiograph, including the RRA, ARA and Cobb analysis of the patient [19] for each examination.

Treatment intervention

**Figure 6:**
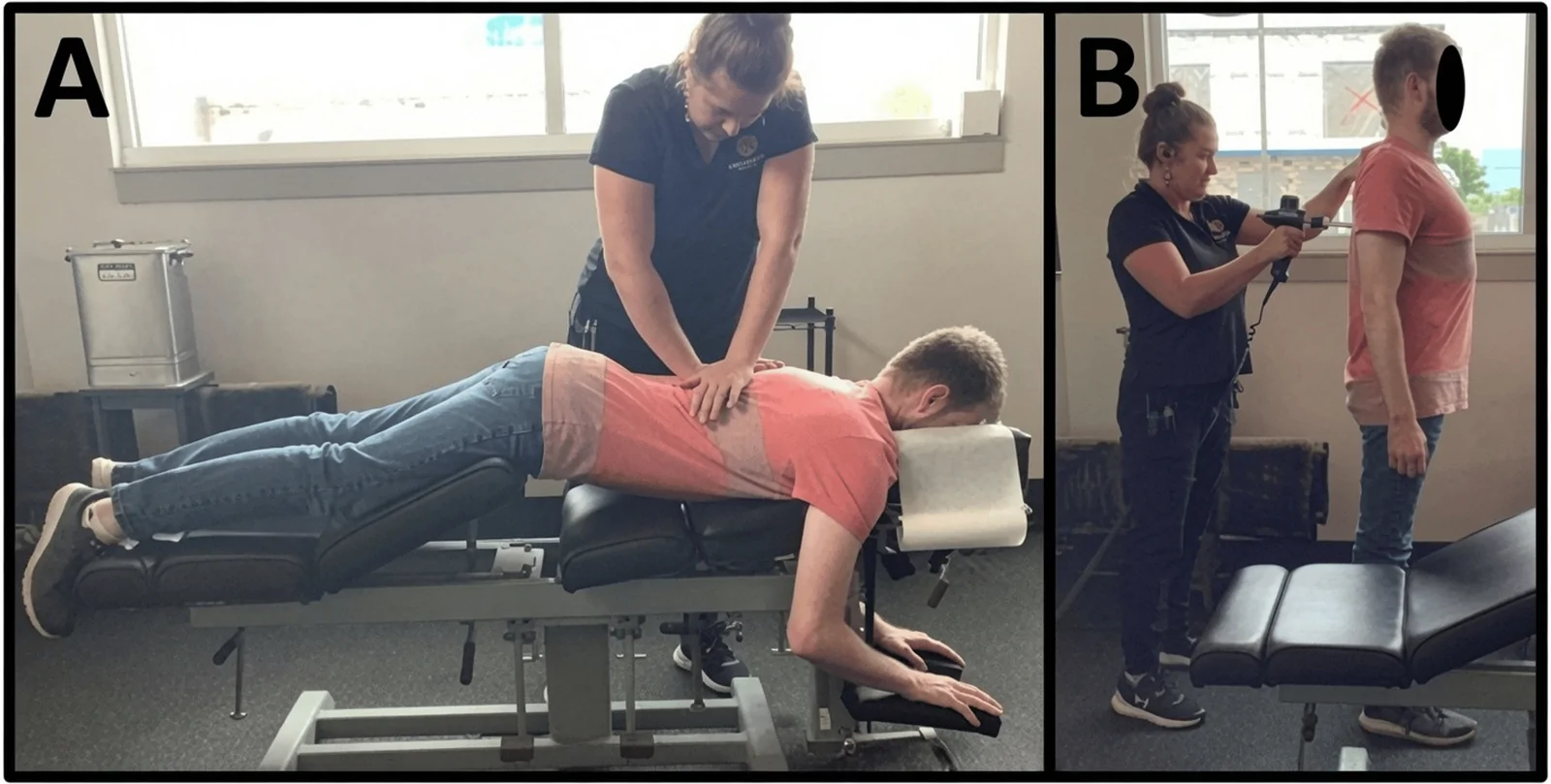
Patient full spine mirror image positioning for drop-table and instrumented postural adjustment. Sagittal mirror image® chiropractic adjustments performed in a) prone position using a drop table and b) standing using an IMPAC pro-Arthrostim® Instrument.

**Figure 7:**
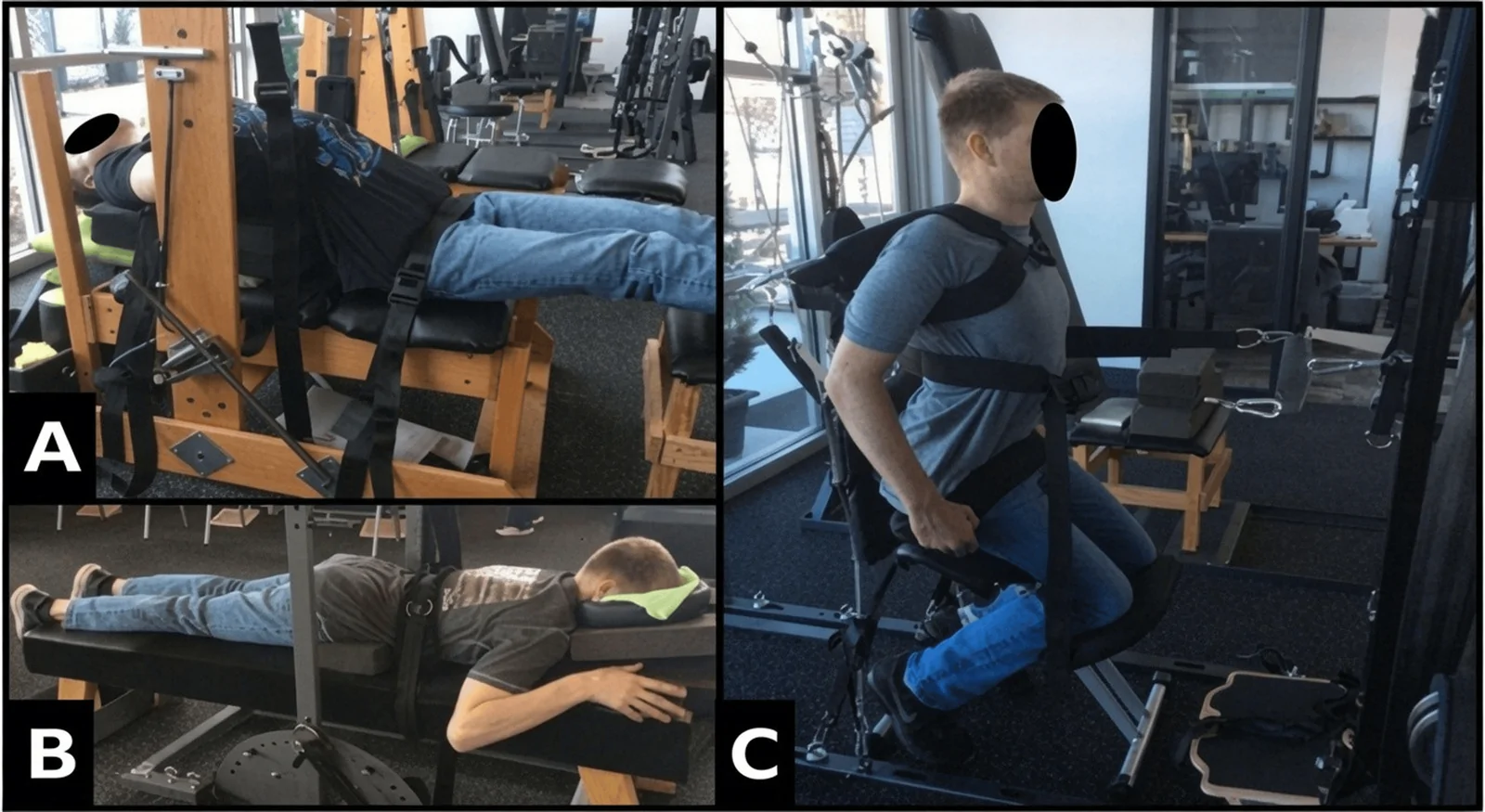
Full spine supine, prone, and seated mirror image thoracic traction. Sagittal mirror image® mechanical traction performed in A) supine, B) prone, and C) seated positions In A, the patient is placed in a supine traction device with the femur heads strapped down with a firm strap and a posterior-anterior soft foam strap across the lower thoracic spine to induce anterior translation and extension. An additional support strap is placed on the upper rib cage to induce some shear forces. In B, the patient is placed prone with a strap across the lower thoracic/upper lumbar spine to cause additional anterior thoracic translation. In C, the patient is in the seated position with a support strap around the shoulder girdle bilaterally to induce extension and prevent flexion; the primary strap was applied at the lower thoracic spine from posterior to anterior, and the pelvis was restrained to induce anterior translation of the thorax and ribcage and extension. The patient was started at 3-5 minutes and progressed to patient tolerance up to 15 minutes of sustained traction force for each traction position.

**Figure 8:**
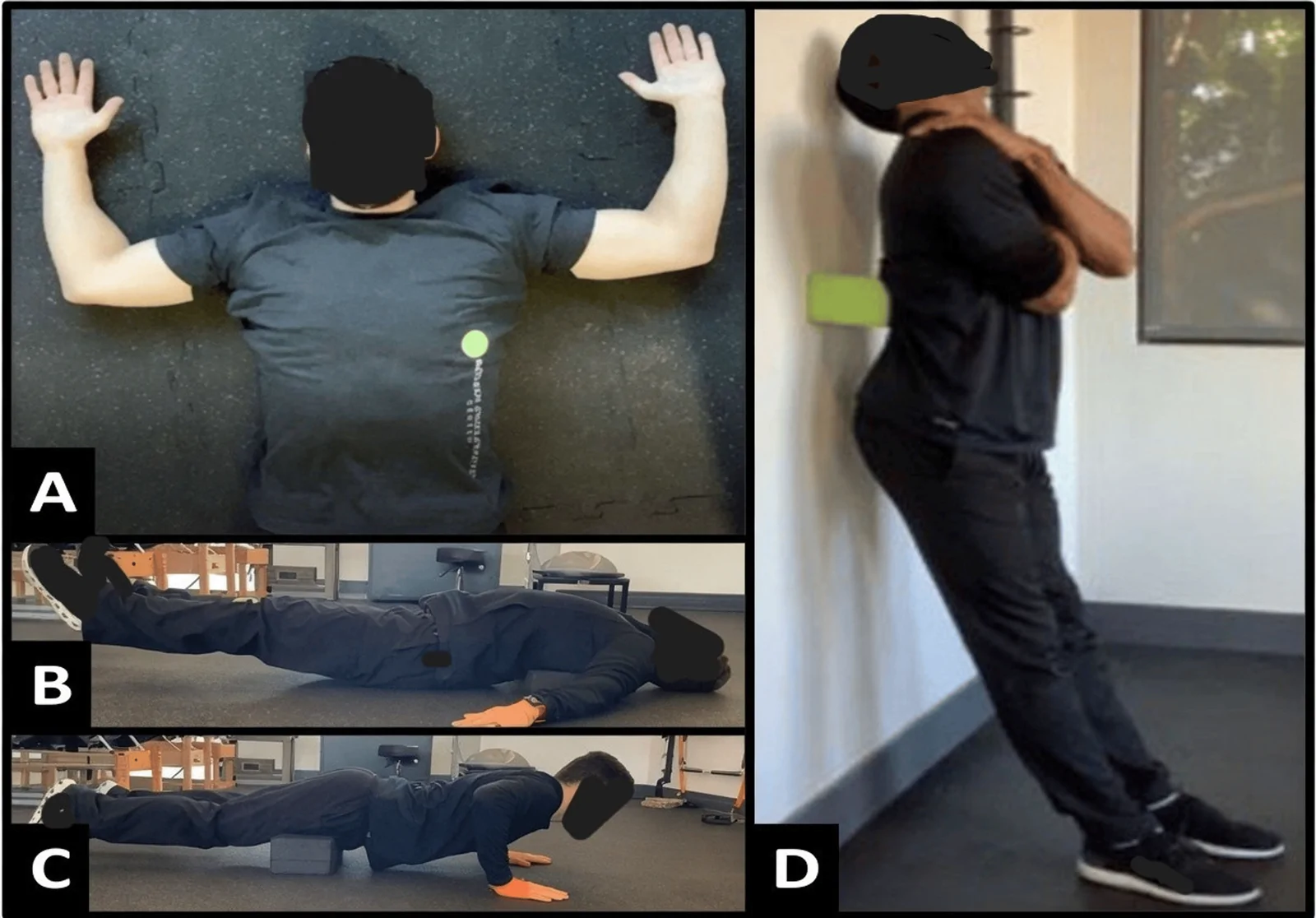
Whole body spine and postural strengthening exercises to minimize thoracic hyperkyphosis. Sagittal mirror image® therapeutic exercises including: A) Floor Angels B) leg raises while supine C) prone Superman exercises D) standing exercises against a wall. In A) The patient was placed supine and was instructed to abduct and retract the shoulders, causing a reduction in the upper thoracic kyphosis. In B) the patient is placed supine with a small foam block placed behind the lower thoracic spine, and the legs are raised to tolerance to strengthen the abdominal and core muscles while causing anterior thoracic translation. In C) patient is placed prone with a foam block under the pelvis to increase thoracic anterior translation, and the patient is instructed to perform the “superman” position with the arms above the head and the upper torso extended to strengthen and flatten the thoracic spine musculature. In D), the patient is placed standing with the feet anterior to the pelvis; a large block is placed at the lower lumbar spine and the upper torso is extended to increase anterior thoracic translation and cervical extension.

The patient was treated 42 times in-office over 14 weeks. The patient received Chiropractic Biophysics® CBP® Mirror Image® (MI®) adjustments using a chiropractic drop table (Figure 6A) and instrument adjusting using an IMPAC ProArthroStim® Instrument (I.M.P.A.C. Inc., Salem, OR, USA) (Figure 6B) [20]. The MI® therapies involve positioning the patient in a corrected or over-corrected postural position (-TzP, +TzT, -RxT, -TzH). Sagittal MI® mechanical traction was performed in supine (Figure 7A), prone (Figure 7B), and seated positions (Figure 7C). MI® therapeutic exercises included prone “angel’ or ‘superman” thoracic flattening and extension strengthening exercises. (Figure 8A), also, leg raises while supine on the floor with a medium CBP® MI® Thoracic support block (CBP Seminars, Inc., Eagle, ID, USA) placed as a fulcrum at the apex of the thoracic kyphosis (Figure 8B), prone Superman exercises on the floor with a large CBP® MI® Block under the pelvis to induce +TzT and -RxT (Figure 8C), and standing exercises with back facing the wall and a block creating a fulcrum at the apex of the thoracic kyphosis (Figure 8D). At-home therapies were prescribed for the patient, including MI® mechanical traction using the Thoracic DennerollTM and therapeutic exercise using CBP® traction blocks [21-23].

A re-exam was performed following 24 CBP® MI spinal rehabilitation visits over eight weeks. The patient rated his MBP and LBP 0/10 on the NRS. The patient scored 0% on his re-exam ODI, indicating no disability due to LBP [24]. Re-exam SF-36 scores improved with: pain at 50%, loss of function due to emotional issues at 60.5%, and energy at 60%. Re-exam posture analysis showed improvement in sagittal posture and balance. Re-exam sagittal radiographic analysis showed improvement in sagittal balance and hyperkyphosis of the thoracic spine with the following measurements: ARA T2-T11 measuring 54.9°, ARA T4-L2 (max ARA) measuring 66.8°, Cobb T4-T12 measuring 64.1°, Cobb T4-L2 (max Cobb) measuring 74.0°, and Tz T12-S1 measuring - 77.4 mm. The treatment rationale for re-examination is prior studies of CBP referring to re-evaluation between 24 and 36 treatments.

A post-treatment exam was performed following an additional 18 CBP® MI spinal rehabilitation visits (42 visits total) over another 6 weeks (14 weeks total). The patient maintained his MBP and LBP at 0/10 on the NRS. The patient scored 0% on his post-treatment ODI, indicating no disability due to LBP. Post-treatment SF-36 scores were further improved and within normal limits with: BP at 100, EWB at 68, EF at 90, and GH at 100, all meeting the MCID for each category. Post-treatment posture analysis showed continued improvement in sagittal posture and balance. Post-treatment sagittal radiographic analysis showed further improvement in sagittal balance and hyperkyphosis of the thoracic spine with the following measurements: ARA T2-T11 measuring 43.8°, ARA T4-L2 (max ARA) measuring 51.3°, Cobb T4-T12 measuring 62.7°, Cobb T4-L2 (max Cobb) measuring 64.6°, Tz T12-S1 measuring -64.1 mm, and TZ T1-T12 measuring 40.3 mm.

A 10-month long-term follow-up exam was performed following an additional 23 maintenance visits and at-home therapies (65 visits total) over another 42 weeks (66 weeks total), including MI® postural adjustments, MI® spine traction and MI® postural exercises. The patient maintained his MBP and LBP at 0/10 on the NRS. The patient maintained 0/100 on his ODI, indicating no disability due to LBP. 10-month long-term follow-up SF-36 maintained scores WNL. Long-term follow-up posture analysis showed continued improvement in sagittal posture and balance (Figure 1). Post-treatment sagittal radiographic analysis showed further improvement in sagittal balance and hyperkyphosis of the thoracic spine with the following measurements: ARA T2-T11 measuring 42.0°, ARA T4-L2 (max ARA) measuring 50.7°, Cobb T4-T12 measuring 56.1°, Cobb T4-L2 (max Cobb) measuring 59.0°, and TZ T1-T12 measuring 12.7 mm.

At 28-month follow-up, the patient was re-examined, the original subjective and objective outcome measures were acquired, and radiography was updated. The patient reported very mild and occasional mid back and low back pain (NRS 1-2/10), NDI measured 0%, ODI measured 8% (very mild), and the SF-36 results found physical function (100%), physical limitation due to pain (100% function), limitation of emotional function due to the condition (100% function), Energy/Fatigue (80%), emotional wellbeing (76%), social functioning (100%), pain (100%), general health (100%) [25]. The 28-month sagittal radiographic analysis showed some slight regression in sagittal balance and hyperkyphosis of the thoracic spine with the following measurements: ARA T2-T11 measuring 52.2°, ARA T4-L2 (max ARA) measuring 61.6°, Cobb T4-T12 measuring 61.6°, Cobb T4-L2 (max Cobb) measuring 75.0°, and Tz T12-S1 measuring -87.1 mm (Figure 7A-7C).

Postural findings demonstrated improved hyperkyphosis and posterior translation, most predominantly at post-treatment evaluation and 10-month follow-up. 28-month follow-up found some return toward baseline in both the postural assessment and radiographs. This could be attributed to a lack of frequency or duration of therapies per week coupled with the patient's new job in law enforcement, which required wearing a large belt in a seated position for many hours on end. The patient’s perspective was one of very high satisfaction with the treatment, and he felt the improved posture and self-confidence contributed to his new employment in law enforcement.

## Discussion

This case documents the successful treatment of a male who suffered from chronic consequences of abnormal spinal alignment due to Scheuermann’s Disease. Following an in-office treatment program coupled with a home-therapy program, it was found that the patient improved in both subjective and objective outcome measures and had improved posture and less spinal misalignment. His condition was initially associated with minimal pain, but the patient had increasing BDD due to the worsening of his posture prior to treatment. The postural and structural abnormalities prevented the patient from acceptance into the USCG [6]. Following treatment, structural changes in both posture and spine alignment improved, as did the patient’s HRQoL outcome measures. Although the patient did not continue to pursue enlistment in the USCG, he was able to be a successful law enforcement officer and exhibited no signs of BDD or any biopsychosocial impact from his posture and spine abnormality. 10-month and 28-month long-term follow-up evaluations found successful improvement in outcome measures.

Recent studies have investigated the association between postural abnormalities and changes in the biopsychosocial effects of body perception-related problems in patients [26-28]. Body dysmorphic disorder (BDD) has been previously defined as a preoccupation with perceived defects in physical appearance; this often leads to significant distress and impairs function. BDD has been associated with major depression, social anxiety disorders, and obsessive-compulsive disorders, and recent studies have found lifetime rates of major depression as high as 76% [29]. Our patient experienced shame, embarrassment and low self-esteem due to his postural abnormality, which may have contributed to poor quality of life following his denial to serve in the USCG.

Altered posture and spine alignment have multiple abnormal consequences for human upright spine health. Sensorimotor alterations, biorheological effects of abnormal tissue loading, mechanoreceptive aberration resulting in proprioceptive deviations, pain, alterations in range of motion, and reduced HRQoL measures have all been reported in the biomedical literature. The literature has extensive studies regarding abnormal posture and spine alignment and altered health outcome measures. Conversely, there are only a few articles regarding successful treatment of altered hyperkyphotic thoracic spine structure with long-term subjective and objective outcome measures reporting sustained improvement.

Prior studies using CBP® Structural and orthopedic rehabilitation techniques have reported improvement in postural abnormalities and spine misalignment. These studies included basic science and modeling investigations, case series and cohort studies, systematic reviews, and randomized and non-randomized case-control studies, have consistently demonstrated improvement across multiple parameters [21,30-34]. It has been reported that improvement in postural abnormalities and altered spine alignment improves short- and long-term outcomes, and this case report adds to that evidence. There is a gap in the research and economical knowledge of the medical literature concerning treatment for early spine and postural abnormalities, and the biopsychosocial impact of treatment versus no treatment for these conditions. Future studies should determine the impact of postural treatment on not only pain and suffering, but also the mental health and self-perception that could improve following postural and spine structural treatment focused on all of the neuromuscular tissues involved in these spine alignment abnormalities.

Radiography is necessary to obtain baseline information regarding the patient’s spine-related conditions [34-38]. Recent studies confirmed that withholding radiographic analysis from patients with only poor posture, regardless of pain and disability, is negligent care. The patient had significant alteration in disc space, unexpected osteophytes at several levels, and advanced disc degeneration that would not be consistent with his age. Bio-rheological and biomechanical studies can explain the alteration in expected normal bone and soft tissue development using principles such as Wolff’s and Davis’ law, which explain degeneration in hard and soft tissues due to abnormal tissue loading [39-41]. Future investigations can investigate these alterations due to abnormal loads, and future studies could also focus on the neuromechanical and neurophysiological consequences of abnormal posture and abnormal spine alignment [42,43].

Without X-ray confirmation of the severity of the condition, application of any rehabilitation would have been considered guessing and not within the standard of care and current evidence for the necessity of radiographic analysis of abnormal posture and BDD, such as was found in this patient. When physicians have inadequate diagnostic patient condition information for triage, the likelihood of making the wrong decisions increases [44,45]. Failure to properly diagnosis and treat these conditions can increase the risk of developing adult spine deformity (ASD), sharply increasing disability risk [46-48]. Simple, future, larger studies should study the measured effect of abnormal posture and spine alignment in BDD and potential conservative treatment options to lessen perceived and actual deformity as well as improve quality of life, potential disability reduction, as well as changes in mental health. Physicians, clinicians and therapists would be wise to assess for postural deviations both perceived and measured with objective devices such as photographs and spine radiographs. Patient perception of their own posture is rarely asked about and could provide significant information regarding patient self-perception of body dysmorphic disorders and physical performance and ability. 

Limitations of this study are the reporting of results on only one patient; however, these results are consistent with prior literature. This limitation of a single patient is inherent in the case report. The authors wish only to report an interesting case and make no assertions of efficacy of any of the treatments. We understand that larger, more robust studies would be necessary to make any definitive efficacy conclusions. The single subject is a limitation. Statistics generally are not considered significant until at least 40 subjects, so the lack of a control subject/group is a limitation. Limitations also include the relatively short long-term follow-up (1 year); longer-term (5-year or 10-year) follow-up studies would provide better confirmation of long-term benefits. Additionally, the subjective nature of PROs could be improved with additional functional testing and longer-term follow-up of the findings.

## Conclusions

The beneficial improvements following CBP® corrective rehabilitation were reported, and a reduction in thoracic deformity, MBP, mild disability, and decreased HRQoL and abnormal PROs due to abnormal posture, BDD and rejection by the USCG for this abnormality. The patient initially had significant mental distress due to his hyperkyphosis and deformity. Following treatment and posture improvement, the patient's mental health and distress improved. The patient’s confidence and self-perception improved following short- and long-term treatment with CBP® and subsequent postural and structural change. A simple multi-modal conservative treatment protocol found that treating Scheuermann’s Disease may improve pain and deformity conservatively and potentially reduce pharmacologic and more invasive interventions. Improvement in spinal alignment and posture may result in long-term reduction or resolution of Scheuermann’s hyperkyphosis and improved HRQoL. This case shows the need for more conservative research involving spinal biomechanics and rehabilitation in patients with spine pain and abnormal spine alignment.
